# Post marketing safety assessment of the novel postpartum depression drug, Zuranolone: evidence from real-world pharmacovigilance analysis based on the FDA adverse event reporting system

**DOI:** 10.3389/fpsyt.2025.1517773

**Published:** 2025-08-15

**Authors:** Duoqin Huang, Zixin Luo, Xi Gong, Kang Zou, Yu Peng, Shaoying Zeng

**Affiliations:** ^1^ The First Clinical Medical College, Gannan Medical University, Ganzhou, Jiangxi, China; ^2^ Department of Psychology, Gannan Medical University, Ganzhou, Jiangxi, China; ^3^ Department of Critical Care Medicine, the First Affiliated Hospital of Gannan Medical College, Ganzhou, Jiangxi, China; ^4^ Department of Gynecology and Obstetrics, People’s Hospital of Ningxiang City, Ningxiang, Hunan, China; ^5^ Department of Obstetrics and Gynecology, the First Affiliated Hospital of Gannan Medical University, Ganzhou, Jiangxi, China

**Keywords:** Zuranolone, FAERS database, safety analysis, postpartum depression, signal mining, adverse events

## Abstract

**Objective:**

Zuranolone, the latest oral medication for postpartum depression, was approved in the United States in August 2023. Due to its pharmacokinetic characteristics and rapid onset of action, it is hailed as a breakthrough and enhanced version of the drug. However, there is limited information on adverse drug reactions associated with its use. The primary objective of this study is to assess the post-marketing safety of Zuranolone. This study utilizes the FAERS database to analyze the safety of Zuranolone and provide a reference for clinical safety.

**Methods:**

Data on Zuranolone were collected from the FAERS database, covering the period from the third quarter of 2023 to the second quarter of 2024. Disproportionate analysis was used to quantify adverse drug reaction signals associated with Zuranolone and to detect risk signals from the data in the FAERS database. The Reporting Odds Ratio (ROR), Proportional Reporting Ratio (PRR), Bayesian Convolutional Probabilistic Neural Network (BCPNN), and Multi-Item Gamma Poisson Shrinker (MGPS) were used collectively to detect risk signals.

**Results:**

This study identified 154 reports primarily suspecting Zuranolone and 426 adverse drug events from a total of 1,626,204 adverse event (AE) reports. A total of 142 Preferred Terms (PTs) were identified across 18 System Organ Classes (SOCs). Most reports originated from the United States, with various health professionals and consumers being the main reporters. Adverse reactions following Zuranolone administration predominantly involved Nervous system disorders and Psychiatric disorders. Specific adverse reactions included Somnolence, Dizziness, Fatigue, Sedation, Suicidal ideation, Tremor, Feeling abnormal, Headache, Anxiety, and Nausea. The onset of AEs related to Zuranolone was not prolonged (average onset time of 4 days, with a median onset time of 2 days). Compared to Brexanolone, Zuranolone’s adverse reactions were more focused on nervous system diseases, while the latter was primarily associated with psychiatric disorders, General disorders and administration site conditions, and Injury, poisoning and procedural complications. Some adverse reactions related to Zuranolone were reported frequently but were not documented in the prescribing information, including Insomnia, Vertigo, Vision blurred, Migraine, and Muscle twitching.

**Conclusion:**

This study revealed potential AEs of Zuranolone, confirming known safety information about Zuranolone, providing comprehensive data for medical practice and public health decision-making, and laying the foundation for further clinical research. It also provides more comprehensive and updated evidence for the clinical safety of Zuranolone.

## 1 Introduction

Postpartum Depression (PPD) is a common mental disorder that typically occurs 4 to 6 weeks after childbirth and is closely associated with negative impacts on the mother and family, as well as serious adverse consequences for the infant’s growth and development, such as low birth weight, delayed development, and emotional disorders (1). The symptom complex of PPD is characterized by low mood, loss of interest and pleasure, reduced energy, and is accompanied by other psychological and somatic symptoms (2). The prevalence of PPD appears notably elevated, with an overall rate approximating 14%, particularly pronounced in developing countries. In China, the prevalence rate of postpartum depression exceeds 20% (3). Therefore, PPD, as a global serious public health issue, has garnered significant attention. Treatment methods for PPD commonly include pharmacological treatment, physical therapy, evidence-based psychotherapy, neuromodulation, hormonal therapy, and lifestyle adjustments (4).

The current pharmacological treatment strategies for PPD are mostly adapted from treatment strategies for non-perinatal adult moderate to severe depression, and are therefore also applicable to moderate to severe depression outside the perinatal period, including selective serotonin reuptake inhibitors (SSRIs), serotonin and norepinephrine reuptake inhibitors (SNRIs), and tricyclic antidepressants (TCAs) (5). Among these, SSRIs remain the first-line treatment options for PPD. However, some studies suggest their limited efficacy in specific cases. However, the evidence for the use of these antidepressants in the treatment of PPD is limited by the small number of randomized clinical trials, low sample power, and limited efficacy based on current evidence (6). In 2019, Brexanolone was FDA approved as a specific antidepressant and treatment for PPD, which can rapidly alleviate perinatal depressive symptoms and has shown good results in patients with moderate to severe PPD, gradually being applied clinically. However, its high cost, severe adverse reactions, and limited accessibility have limited its use in clinical practice (7).

Zuranolone, a new anti-PPD drug that was launched in 2023, is an enhanced version of Brexanolone, optimizing the selectivity for synaptic and extra synaptic GABA receptors, as well as the pharmacokinetic characteristics of oral administration, overcoming the major drawback of Brexanolone requiring 60 hours of intravenous injection (8). It also continues the advantages of rapid onset and short course of treatment. Recent studies have shown that zuranolone is more effective in treating postpartum depression than most first-line medications, such as SSRIs, used in many countries. On day 15 after treatment, the improvement in symptoms of PPD patients treated with zuranolone was greater than that of patients treated with SSRIs, and significant greater improvement was also observed at later time points, with the maximum mean difference on day 45 (9). In addition, relevant economic models have also indicated that compared with SSRIs, zuranolone is a more cost-effective therapy for the treatment of adult PPD (10). However, it should be noted that the use of Zuranolone aftermarket launch has raised some clinical safety issues. However, the current evidence on safety is almost based on a small number of clinical trials (11). At the same time, the adverse reaction warnings in the standard prescribing information for Zuranolone, including suicidal thoughts, fetal toxicity, and central nervous system inhibitory toxicity, are limited by the limitations of pre-market research and the short time since market launch, and cannot provide comprehensive help for clinical medication (12). Pharmacovigilance analysis is a key data source for assessing adverse drug reactions (ADRs) in the real world. As Zuranolone is gradually widely used in clinical settings, it is necessary to conduct post-marketing assessment of its safety using data mining techniques.

The FDA Adverse Event Reporting System (FAERS) is a public pharmacovigilance database designed to facilitate the FDA’s post-marketing safety monitoring of drugs and therapeutic products (13). Since the official launch of Zuranolone in 2023, there has been no systematic report on its adverse reactions. There is a lack of data evidence for the actual clinical safety assessment of Zuranolone. In this study, we retrospectively mine and analyze relevant adverse reaction reports through data mining to explore its safety in the real world, evaluate relevant risk factors, and provide a reference for post-marketing safety assessment of drugs. Our research results may help doctors and health decision-makers to monitor adverse reactions, thereby promoting the rational use of clinical drugs.

## 2 Methods

### 2.1 Data source and collection

The FAERS database is a large pharmacovigilance database that contains information on adverse events and medication errors reported by healthcare professionals or patients from countries around the world (14). The data from the FAERS database is divided into seven modular arrays, including demographic and administrative information (DEMO), adverse drug reaction information (REAC), patient outcome information (OUTC), drug information (DRUG), dates of drug therapy start and end (THER), reporter information (RPSR), and indication for use/diagnosis (INDI) (15). These data are based on the International Safety Reporting Guidelines issued by the ICH (E2B), and adverse events are coded according to the Medical Dictionary for Regulatory Activities (MedDRA). These drugs can be categorized as primary suspect drugs, secondary suspect drugs, concomitant medications, and interacting medications. As the FAERS database is publicly available, it does not require ethical approval from an ethics committee or potential informed consent.

### 2.2 Data procedures

**Figure 1:**
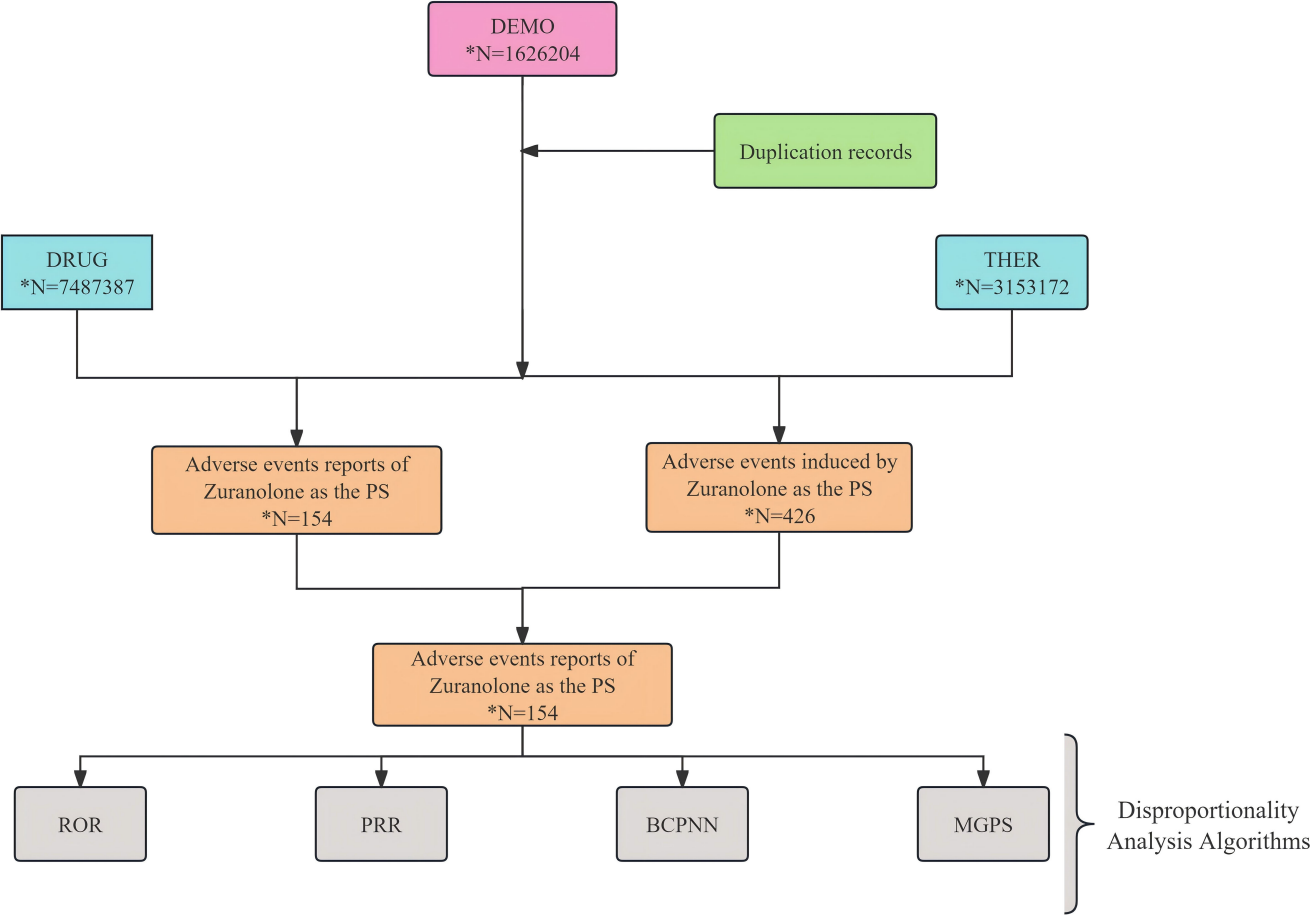
Flow diagram of this study (DEMO, demographic and administrative information; DRUG, drug information; THER, dates of drug therapy start and end; PS, primary suspect drug).

Referring to the recommendations of the U.S. FDA and considering the official market launch time of Zuranolone, we filtered data from all quarters in the FAERS database between the third quarter of 2023 and the second quarter of 2024 for analysis. The search names included the generic and trade names of the drug, such as “Zuranolone,” “Zurzuvae (the trade name for Zuranolone),” and “SAGE-217.” Since a report may have multiple drugs and adverse drug reactions or involve duplicate reports of the same adverse event case, we removed older duplicate records and selected the most recent FDA_DT with the same CASEID. A total of 154 reports were obtained where Zuranolone was the primary suspect drug (PS), and the flowchart is shown in **Figure 1**. All analyses of AE reports for Zuranolone were based on reports where it was the primary suspect drug in the ADE. We identified them at the System Organ Class (SOC) and Preferred Term (PT) levels.

### 2.3 Statistical analysis

Disproportionate analysis is a pharmacovigilance method that uses a case/non-case design to identify a drug that is more frequently associated with a given adverse event than other drugs (16). In this paper, the association between Zuranolone and AEs was determined using four algorithms: Reporting Odds Ratio (ROR), Proportional Reporting Ratio (PRR), Bayesian Convolutional Probabilistic Neural Network (BCPNN), and Multi-Item Gamma Poisson Shrinker (MGPS) (17), as seen in **Table 1**. To enhance the robustness of these methods and reduce the likelihood of false positives, we chose to consider an AE signal as positive if it met the criteria of the all four algorithms. Among the positive signals, we then compared them again with the drug package insert to select any significant AEs that were not listed in the package. In analyzing the onset time of adverse reactions, we calculated the time interval between EVENT_DT (the date of the adverse event) and START_DT (the start date of Zuranolone use). If there were input errors (EVENT_DT earlier than START_DT) or missing date entries (missing exact days or months), these data were excluded from the analysis. All analyses were conducted using R software (version 4.2.3).

**Table 1:** Equations and criteria for the four disproportionality analysis algorithms.

Algorithms	Equation	Criteria
ROR	ROR=(a/c)/(b/d) =ad/bc	Lower limit of 95% *CI*>1
	SE (ln*ROR)*=(1/a+1/b+1/c+1/d) ^0.5	N≥3
	95% *CI*=e^ln (ROR) ± 1.96(1/a+1/b+1/c+1/d) ^0.5^	
PRR	PRR=[a(a+b)]/[c/(c+d)]	PRR≥2
	SE (ln*PRR)*=(1/a-1/(a+b) +1/c-1/(c+d)) ^0.5	N≥3
	95% *CI*=e^ln (PRR) ± 1.96(1/a-1/(a+b) +1/c-1/(c+d)) ^0.5^	χ^2^≥4
BCPNN	IC=log_2_[a(a+b+c+d)/((a+c) (a+b))]	E(IC)>0
	E(IC)=log_2_[(a+γ11) (a+b+c+d+α) (a+b+c+d+β)/((a+b+c+d+γ) (a+c+β1) (a+b+α1))]	IC025 > 0
	V(IC)=1/(ln2)^2{[((a+b+c+d)-α+γ-γ11)/((a+γ11)(1+a+b+c+d+γ))]+[(a+b+c+d-a-b+α-α1)/((a+b+α1)(1+a+b+c+d+α))]+[(a+b+c+d-a-c+β-β1)/((a+c+β1)(1+a+b+c+d+β))]}	
	IC-2SD= E(IC) -2V(IC)^0.5	
MGPS	EBGM=a(a+b+c+d)/((a+c) (a+b))	EBGM05>2
	EBGM05=e^ln (EBGM)-1.64(1/a+1/b+1/c+1/d)^0.5^	

a, number of reports containing both the target drug and target adverse drug reaction; b, number of reports containing other adverse drug reaction of the target drug; c, number of reports containing the target adverse drug reaction of other drugs; d, number of reports containing other drugs and other adverse drug reactions. 95%CI, 95% confidence interval; N, the number of reports; χ2, chi-squared; IC, information component; IC025, the lower limit of 95% CI of the IC; E(IC), the IC expectations; V(IC), the variance of IC; EBGM, empirical Bayesian geometric mean; EBGM05, the lower limit of 95% CI of EBGM.

## 3 Results

### 3.1 General characteristics

We identified 154 reports with Zuranolone as the primary suspect drug and 426 adverse drug events (ADEs). The average age of patients with available data was 31.6 years (range, 22 to 45 years). Since Zuranolone is primarily used for the treatment of postpartum depression, patient gender was not specifically considered in this analysis. All patient data were reported from the United States. 84 healthcare professionals (54.5%), including health professionals, physicians, and pharmacists, and 70 consumers (45.5%) reported the case data. Perinatal depression was the most frequently reported indication, followed by major depressive disorder. For the use of Zuranolone, 154 primary suspect drug reports accounted for the majority. Serious outcomes included life-threatening, hospitalization, and other significant consequences. The top five other antidepressants associated with Zuranolone when it was a secondary suspect drug were Ativan, Fluoxetine, Zoloft, Lexapro, and Sertraline. The demographic and clinical characteristics of Zuranolone-related cases are shown in **Table 2**.

**Table 2:** Clinical characteristics of reports with Zuranolone from the FAERS database.

Characteristics	Case, n (%)	Data availability, n (%)
Reported role of Zuranolone		
Primary	154	*
Age, Median	31.6 (22~45)	77 (50.0)
≤30	38 (49.4)	
30~40	32 (41.5)	
>40	7 (9.1)	
Reporting country		154 (100)
United States	154 (100)	
Type of reporter		154 (100)
Consumer	70 (45.5)	
Health professional	39 (25.3)	
Physician	32 (20.8)	
Pharmacist	13 (8.4)	
Reporting year		154 (100)
2023	1 (0.6)	
2024	153 (99.4)	
Indication		147 (95.5)
Major depression	1 (0.7)	
Perinatal depression	95 (64.6)	
Unknown indication	51 (34.7)	
Serious outcome of event		20 (13.0)
Life-threatening	1 (5.0)	
Hospitalization	5 (25.0)	
Other Serious Outcome	14 (70.0)	

*All reporting analyses of AEs for Zuranolone were based on their reports of the PS;A report may have one or more outcome of events.

### 3.2 Signal detection

#### 3.2.1 Signal detection results

Based on 154 primary suspect drug reports, a total of 426 Zuranolone adverse reaction reports were screened, involving 142 PTs in 18 SOC.

#### 3.2.2 Signal detection of Zuranolone at the SOC level

This study identified 18 signals for Zuranolone at the SOC level through signal mining, with the signal strength reported in **Table 3**. Two important SOC met the positive signal selection criteria, which were Nervous system disorders and Psychiatric disorders. Among them, the most frequently reported SOC was Nervous system disorders (n=179). Additionally, Ear and labyrinth disorders were important SOCs that met the criteria for at least one of the four indicators. However, adverse events related to the systems of Ear and labyrinth disorders, Eye disorders, social circumstances, Metabolism and nutrition disorders, Cardiac disorders, and Vascular disorders were not mentioned in the prescribing information.

**Table 3:** Signal strength of reports of Zuranolone at the SOC level.

System organ class	Case reports	ROR (95%Cl)	PRR (X^2^)	EBGM (EBGM05)	IC (IC025)	Signal
Nervous System Disorders	179	9.43 (7.78 - 11.44) *	5.89 (782.32) *	5.89 (5.01) *	2.56 (0.89) *	Yes
Psychiatric Disorders	92	5.16 (4.1 - 6.5) *	4.26 (242.03) *	4.26 (3.51) *	2.09 (0.42) *	Yes
General Disorders and Administration Site Conditions	61	0.8 (0.61 - 1.05)	0.83 (2.68)	0.83 (0.66)	-0.27 (-1.95)	No
Gastrointestinal Disorders	17	0.46 (0.28 - 0.74)	0.48 (10.58)	0.48 (0.32)	-1.07 (-2.74)	No
Injury, Poisoning and Procedural Complications	13	0.2 (0.11 - 0.34)	0.22 (41.33)	0.22 (0.14)	-2.18 (-3.85)	No
Musculoskeletal And Connective Tissue Disorders	11	0.46 (0.26 - 0.85)	0.48 (6.61)	0.48 (0.29)	-1.06 (-2.74)	No
Eye Disorders	11	1.3 (0.72 - 2.37)	1.29 (0.75)	1.29 (0.78)	0.37 (-1.3)	No
Investigations	7	0.26 (0.12 - 0.54)	0.27 (14.96)	0.27 (0.14)	-1.9 (-3.57)	No
Respiratory, Thoracic and Mediastinal Disorders	6	0.29 (0.13 - 0.65)	0.3 (10.25)	0.3 (0.15)	-1.73 (-3.41)	No
Ear And Labyrinth Disorders	6	3.23 (1.44 - 7.23) *	3.2 (9.1) *	3.2 (1.63)	1.68 (0)	No
Reproductive System and Breast Disorders	5	2.12 (0.88 - 5.13)	2.11 (2.94)	2.11 (1.01)	1.08 (-0.59)	No
Metabolism And Nutrition Disorders	5	0.59 (0.25 - 1.44)	0.6 (1.36)	0.6 (0.29)	-0.74 (-2.41)	No
Surgical And Medical Procedures	5	0.73 (0.3 - 1.75)	0.73 (0.51)	0.73 (0.35)	-0.45 (-2.13)	No
Infections And Infestations	2	0.07 (0.02 - 0.27)	0.07 (25.31)	0.07 (0.02)	-3.78 (-5.45)	No
Vascular Disorders	2	0.26 (0.06 - 1.02)	0.26 (4.33)	0.26 (0.08)	-1.95 (-3.62)	No
Social Circumstances	2	0.96 (0.24 - 3.85)	0.96 (0)	0.96 (0.3)	-0.06 (-1.73)	No
Skin And Subcutaneous Tissue Disorders	1	0.04 (0.01 - 0.3)	0.04 (21.56)	0.04 (0.01)	-4.48 (-6.16)	No
Cardiac Disorders	1	0.13 (0.02 - 0.95)	0.14 (5.64)	0.14 (0.03)	-2.89 (-4.56)	No

*Statistically significant signals in the algorithm; All reporting analyses of AEs for Zuranolone were based on their reports of the PS; A report may have one or more outcome of events.

#### 3.2.3 Signal detection of Zuranolone at the PT level

Zuranolone identified 142 signals at the PT level, with 28 positive signals found. Furthermore, there were 103 important PTs that met the criteria for at least one of the four evaluation indicators. Based on the frequency of occurrence and SOC classification, the top 35 frequently occurring and the top 5 frequently occurring in each SOC classification are listed (**Tables 4**, **5**). Sorted by frequency (**Table 4**), the top 10 PTs included Somnolence, Dizziness, Fatigue, Sedation, Suicidal ideation, Tremor, feeling abnormal, Headache, Anxiety, and Nausea, all of which correspond well with existing clinical evidence. High-frequency adverse reactions such as Insomnia, Vertigo, Vision blurred, Migraine, and Muscle twitching were not recorded in the prescribing information. From the SOC classification frequency list (**Table 5**), it can be observed that Nervous system disorders and Psychiatric disorders remain the main SOC where the positive signals of PTs are located. Overall, some ADEs, although not frequent, such as Nervousness, Panic attack, Depressed mood, Cognitive disorder, Initial insomnia, Underdose, Bradyphrenia, Anger, etc., showed significant signal strength and may represent new potential ADE signals. There are also some signals that did not reach the positive signal evaluation criteria of this paper, but two or more of the screening indicators had statistical significance and met the criteria, and such signals are also worth attention and importance.

**Table 4:** Top 35 PTs of Zuranolone ranked by report numbers.

Preferred terms	Case reports	ROR (95%Cl)	PRR (X^2^)	EBGM (EBGM05)	IC (IC025)	Signal
Somnolence	52	49.59 (37.09 - 66.31) *	43.66 (2166.57) *	43.52 (34.13) *	5.44 (3.77) *	Yes
Dizziness	36	13.67 (9.72 - 19.24) *	12.6 (386.73) *	12.59 (9.46) *	3.65 (1.98) *	Yes
Fatigue	21	3.61 (2.33 - 5.6) *	3.48 (37.7) *	3.48 (2.41) *	1.8 (0.13) *	Yes
Sedation	17	119.58 (73.47 - 194.63) *	114.85 (1903.08) *	113.89 (75.77) *	6.83 (5.16) *	Yes
Suicidal Ideation	14	30.65 (17.98 - 52.24) *	29.67 (387.49) *	29.61 (18.95) *	4.89 (3.22) *	Yes
Tremor	10	11.9 (6.36 - 22.29) *	11.65 (97.45) *	11.64 (6.88) *	3.54 (1.87) *	Yes
Headache	9	2.32 (1.2 - 4.5) *	2.3 (6.64) *	2.3 (1.32)	1.2 (-0.47)	No
Feeling Abnormal	9	7.39 (3.82 - 14.31) *	7.26 (48.67) *	7.25 (4.17 *	2.86 (1.19) *	Yes
Anxiety	8	4.91 (2.44 - 9.88) *	4.83 (24.4) *	4.83 (2.69) *	2.27 (0.6) *	Yes
Nausea	7	1.5 (0.71 - 3.16)	1.49 (1.14)	1.49 (0.8)	0.58 (-1.1)	No
Drug Ineffective	7	0.9 (0.43 - 1.91)	0.91 (0.07)	0.91 (0.48)	-0.14 (-1.81)	No
Brain Fog	7	18.65 (8.83 - 39.38) *	18.36 (114.84) *	18.34 (9.81) *	4.2 (2.52) *	Yes
Insomnia	7	4.57 (2.16 - 9.64) *	4.51 (19.17) *	4.51 (2.41) *	2.17 (0.5) *	Yes
Feeling Drunk	7	228.62 (107.66 - 485.48) *	224.88 (1534.81) *	221.22 (117.81) *	7.79 (6.11) *	Yes
Vertigo	6	18.1 (8.08 - 40.54) *	17.86 (95.44) *	17.84 (9.08) *	4.16 (2.48) *	Yes
Depression	6	5.4 (2.41 - 12.08) *	5.33 (21.17) *	5.33 (2.72) *	2.41 (0.74) *	Yes
Vision Blurred	5	6.52 (2.7 - 15.75) *	6.45 (23.08) *	6.45 (3.08) *	2.69 (1.02) *	Yes
Migraine	5	7.23 (2.99 - 17.47) *	7.16 (26.52) *	7.16 (3.42) *	2.84 (1.17) *	Yes
Memory Impairment	5	5.33 (2.21 - 12.88) *	5.28 (17.38 *	5.28 (2.52) *	2.4 (0.7) *	Yes
Muscle Twitching	4	32.71 (12.21 - 87.67) *	32.41 (121.53) *	32.34 (14.17) *	5.02 (3.34) *	Yes
Nervousness	4	16.96 (6.33 - 45.42) *	16.81 (59.42) *	16.79 (7.36) *	4.07 (2.4) *	Yes
Panic Attack	4	20.45 (7.63 - 54.79) *	20.27 (73.2) *	20.24 (8.87) *	4.34 (2.67) *	Yes
Perinatal Depression	4	958.12 (346.09 - 2652.48) *	949.13 (3540.13) *	886.96 (378.33) *	9.79 (8.0) *	Yes
Therapy Cessation	4	12.79 (4.78 - 34.25) *	12.68 (43.02) *	12.67 (5.56) *	3.66 (1.99) *	Yes
Balance Disorder	4	7.54 (2.82 - 20.19) *	7.48 (22.47) *	7.48 (3.28) *	2.9 (1.23) *	Yes
Visual Impairment	3	3.43 (1.1 - 10.69) *	3.42 (5.13) *	3.41 (1.32)	1.77 (0.1) *	No
Diarrhoea	3	0.62 (0.2 - 1.92)	0.62 (0.7)	0.62 (0.24)	-0.69 (-2.36)	No
Muscular Weakness	3	3.99 (1.28 - 12.41) *	3.97 (6.66) *	3.96 (1.53)	1.99 (0.32) *	No
Depressed Mood	3	8.62 (2.77 - 26.83) *	8.56 (20.04) *	8.56 (3.31) *	3.1 (1.42) *	Yes
Cognitive Disorder	3	10.81 (3.47 - 33.66) *	10.74 (26.49) *	10.73 (4.15) *	3.42 (1.75) *	Yes
Muscle Spasms	3	2.74 (0.88 - 8.54)	2.73 (3.3)	2.73 (1.06)	1.45 (-0.22)	No
Initial Insomnia	3	32.17 (10.32 - 100.28) *	31.95 (89.75) *	31.88 (12.31) *	4.99 (3.32) *	Yes
Underdose	3	6.98 (2.24 - 21.74) *	6.94 (15.25) *	6.94 (2.68) *	2.79 (1.12) *	Yes
Bradyphrenia	3	112.56 (35.99 - 352.05) *	111.78 (326.68) *	110.87 (42.7) *	6.79 (5.11) *	Yes
Anger	3	22.25 (7.14 - 69.33) *	22.1 (60.36) *	22.07 (8.53) *	4.46 (2.79) *	Yes

*Statistically significant signals in the algorithm; All reporting analyses of AEs for Zuranolone were based on their reports of the PS; A report may have one or more outcome of events.

**Table 5:** The top 5 signal strength of reports of Zuranolone ranked by each SOC at the PTs level.

System organ class	Preferred terms	Case reports	ROR (95%Cl)	PRR (X^2^)	EBGM (EBGM05)	IC (IC025)	Signal
Nervous system disorders	Somnolence	52	49.59 (37.09 - 66.31) *	43.66 (2166.57) *	43.52 (34.13) *	5.44 (3.77) *	Yes
	Dizziness	36	13.67 (9.72 - 19.24) *	12.6 (386.73) *	12.59 (9.46) *	3.65 (1.98) *	Yes
	Sedation	17	119.58 (73.47 - 194.63) *	114.85 (1903.08) *	113.89 (75.77) *	6.83 (5.16) *	Yes
	Tremor	10	11.9 (6.36 - 22.29) *	11.65 (97.45) *	11.64 (6.88) *	3.54 (1.87) *	Yes
	Headache	9	2.32 (1.2 - 4.5) *	2.3 (6.64) *	2.3 (1.32)	1.2 (-0.47)	No
Psychiatric disorders	Suicidal Ideation	14	30.65 (17.98 - 52.24) *	29.67 (387.49) *	29.61 (18.95) *	4.89 (3.22) *	Yes
	Anxiety	8	4.91 (2.44 - 9.88) *	4.83 (24.4) *	4.83 (2.69) *	2.27 (0.6) *	Yes
	Insomnia	7	4.57 (2.16 - 9.64) *	4.51 (19.17) *	4.51 (2.41) *	2.17 (0.5) *	Yes
	Depression	6	5.4 (2.41 - 12.08) *	5.33 (21.17) *	5.33 (2.72) *	2.41 (0.74) *	Yes
	Nervousness	4	16.96 (6.33 - 45.42) *	16.81 (59.42) *	16.79 (7.36) *	4.07 (2.4) *	Yes
General disorders and administration site conditions	Fatigue	21	3.61 (2.33 - 5.6) *	3.48 (37.7) *	3.48 (2.41) *	1.8 (0.13) *	Yes
	Feeling abnormal	9	7.39 (3.82 - 14.31) *	7.26 (48.67) *	7.25 (4.17) *	2.86 (1.19) *	Yes
	Drug ineffective	7	0.9 (0.43 - 1.91)	0.91 (0.07)	0.91 (0.48)	-0.14 (-1.81)	No
	Feeling drunk	7	228.62 (107.66 - 485.48) *	224.88 (1534.81) *	221.22 (117.81) *	7.79 (6.11) *	Yes
	Asthenia	2	0.8 (0.2 - 3.22)	0.8 (0.1)	0.8 (0.25)	-0.31 (-1.99)	No
Gastrointestinal disorders	Nausea	7	1.5 (0.71 - 3.16)	1.49 (1.14)	1.49 (0.8)	0.58 (-1.1)	No
	Diarrhoea	3	0.62 (0.2 - 1.92)	0.62 (0.7)	0.62 (0.24)	-0.69 (-2.36)	No
	Vomiting	2	0.7 (0.18 - 2.83)	0.71 (0.25)	0.71 (0.22)	-0.5 (-2.17)	No
	Abdominal discomfort	2	1.62 (0.4 - 6.49)	1.62 (0.47)	1.61 (0.51)	0.69 (-0.98)	No
	Paraesthesia oral	1	10.51 (1.48 - 74.82)	10.48 (8.58)	10.48 (2.03) *	3.39 (1.72) *	No
Injury, poisoning and procedural complications	Underdose	3	6.98 (2.24 - 21.74) *	6.94 (15.25) *	6.94 (2.68) *	2.79 (1.12) *	Yes
	Prescribed underdose	2	12.45 (3.1 - 49.98)	12.4 (20.94)	12.39 (3.87) *	3.63 (1.96) *	No
	Sedation complication	1	37.14 (5.21 - 264.99)	37.06 (34.99)	36.96 (7.14) *	5.21 (3.53) *	No
	Injury	1	3.81 (0.53 - 27.1)	3.8 (2.06)	3.8 (0.74)	1.93 (0.25) *	No
	Fall	1	0.46 (0.06 - 3.27)	0.46 (0.64)	0.46 (0.09)	-1.12 (-2.79)	No
Musculoskeletal and connective tissue disorders	Muscle twitching	4	32.71 (12.21 - 87.67) *	32.41 (121.53) *	32.34 (14.17) *	5.02 (3.34) *	Yes
	Muscular weakness	3	3.99 (1.28 - 12.41) *	3.97 (6.66) *	3.96 (1.53)	1.99 (0.32) *	No
	Muscle spasms	3	2.74 (0.88 - 8.54)	2.73 (3.3)	2.73 (1.06)	1.45 (-0.22)	No
	Mobility decreased	1	1.76 (0.25 - 12.55)	1.76 (0.33)	1.76 (0.34)	0.82 (-0.86)	No
Eye disorders	Vision blurred	5	6.52 (2.7 - 15.75) *	6.45 (23.08) *	6.45 (3.08) *	2.69 (1.02)*	Yes
	Visual impairment	3	3.43 (1.1 - 10.69) *	3.42 (5.13) *	3.41 (1.32)	1.77 (0.1) *	No
	Diplopia	2	11.24 (2.8 - 45.1)	11.19 (18.55)	11.18 (3.49) *	3.48 (1.81) *	No
	Mydriasis	1	18.44 (2.59 - 131.41)	18.4 (16.44)	18.38 (3.55) *	4.2 (2.52) *	No
Investigations	Blood pressure increased	2	1.61 (0.4 - 6.45)	1.6 (0.46)	1.6 (0.5)	0.68 (-0.99)	No
	Heart rate decreased	2	6.1 (1.52 - 24.46)	6.07 (8.48)	6.07 (1.9)	2.6 (0.93) *	No
	Body temperature decreased	1	8.27 (1.16 - 58.88)	8.25 (6.37)	8.25 (1.6)	3.04 (1.37) *	No
	Blood oestrogen increased	1	246.49 (34.03 - 1785.28)	245.91 (239.56)	241.54 (46.07) *	7.92 (6.2) *	No
	Weight increased	1	0.63 (0.09 - 4.47)	0.63 (0.22)	0.63 (0.12)	-0.67 (-2.34)	No
Respiratory, thoracic and mediastinal disorders	Dyspnoea	2	0.58 (0.14 - 2.32)	0.58 (0.6)	0.58 (0.18)	-0.79 (-2.46)	No
	Yawning	1	100.42 (14.01 - 719.74)	100.19 (97.48)	99.46 (19.14) *	6.64 (4.95) *	No
	Throat Tightness	1	7.35 (1.03 - 52.32)	7.33 (5.47)	7.33 (1.42)	2.87 (1.2) *	No
	Oropharyngeal Pain	1	1.25 (0.18 - 8.89)	1.25 (0.05)	1.25 (0.24)	0.32 (-1.35)	No
	Rhinorrhoea	1	1.73 (0.24 - 12.3)	1.73 (0.31)	1.73 (0.33)	0.79 (-0.88)	No
Ear and labyrinth disorders	Vertigo	6	18.1 (8.08 - 40.54) *	17.86 (95.44) *	17.84 (9.08) *	4.16 (2.48) *	Yes
Reproductive system and breast disorders	Heavy Menstrual Bleeding	1	8.75 (1.23 - 62.3)	8.73 (6.84)	8.73 (1.69)	3.13 (1.45) *	No
	Polymenorrhoea	1	66.45 (9.29 - 475.13)	66.3 (64)	65.98 (12.72) *	6.04 (4.36) *	No
	Dysmenorrhoea	1	15.98 (2.24 - 113.87)	15.95 (14)	15.93 (3.08) *	3.99 (2.32) *	No
	Galactostasis	1	4518.97 (469.11 - 43531.83)	4508.36 (3379.77)	3381.52 (508.11) *	11.72 (9.61) *	No
	Menstruation Irregular	1	17.18 (2.41 - 122.4)	17.14 (15.18)	17.12 (3.31) *	4.1 (2.42) *	No
Metabolism and nutrition disorders	Decreased Appetite	1	0.58 (0.08 - 4.1)	0.58 (0.31)	0.58 (0.11)	-0.79 (-2.46)	No
	Dehydration	1	1.41 (0.2 - 10.04)	1.41 (0.12)	1.41 (0.27)	0.49 (-1.18)	No
	Increased Appetite	1	7.39 (1.04 - 52.58)	7.37 (5.51)	7.37 (1.43)	2.88 (1.21) *	No
	Feeding Disorder	1	5.65 (0.79 - 40.21)	5.64 (3.82)	5.64 (1.09)	2.49 (0.82) *	No
	Hypophagia	1	6.19 (0.87 - 44.07)	6.18 (4.34)	6.18 (1.2)	2.63 (0.95) *	No
Surgical and medical procedures	Therapy Cessation	4	12.79 (4.78 - 34.25) *	12.68 (43.02) *	12.67 (5.56) *	3.66 (1.99) *	Yes
	Therapy Change	1	13 (1.82 - 92.56)	12.97 (11.04)	12.96 (2.51) *	3.7 (2.02) *	No
Infections and infestations	Nasopharyngitis	1	0.56 (0.08 - 4)	0.56 (0.34)	0.56 (0.11)	-0.83 (-2.5)	No
	Acute Sinusitis	1	130.35 (18.15 - 936.3)	130.05 (126.84)	128.82 (24.75) *	7.01 (5.31) *	No
Vascular disorders	Hypotension	1	0.9 (0.13 - 6.41)	0.9 (0.01)	0.9 (0.17)	-0.15 (-1.82)	No
	Blood pressure fluctuation	1	4.48 (0.63 - 31.87)	4.47 (2.69)	4.47 (0.86)	2.16 (0.49) *	No
Social circumstances	Loss of personal independence in daily activities	2	3.66 (0.91 - 14.67)	3.64 (3.84)	3.64 (1.14)	1.87 (0.19) *	No
Skin and subcutaneous tissue disorders	Skin disorder	1	3.75 (0.53 - 26.69)	3.74 (2.01)	3.74 (0.72)	1.9 (0.23) *	No
Cardiac disorders	Palpitations	1	1.63 (0.23 - 11.59)	1.63 (0.24)	1.63 (0.31)	0.7 (-0.97)	No

*Statistically significant signals in the algorithm; All reporting analyses of AEs for Zuranolone were based on their reports of the PS; A report may have one or more outcome of events.

### 3.3 Time-to-onset of events

**Figure 2:**
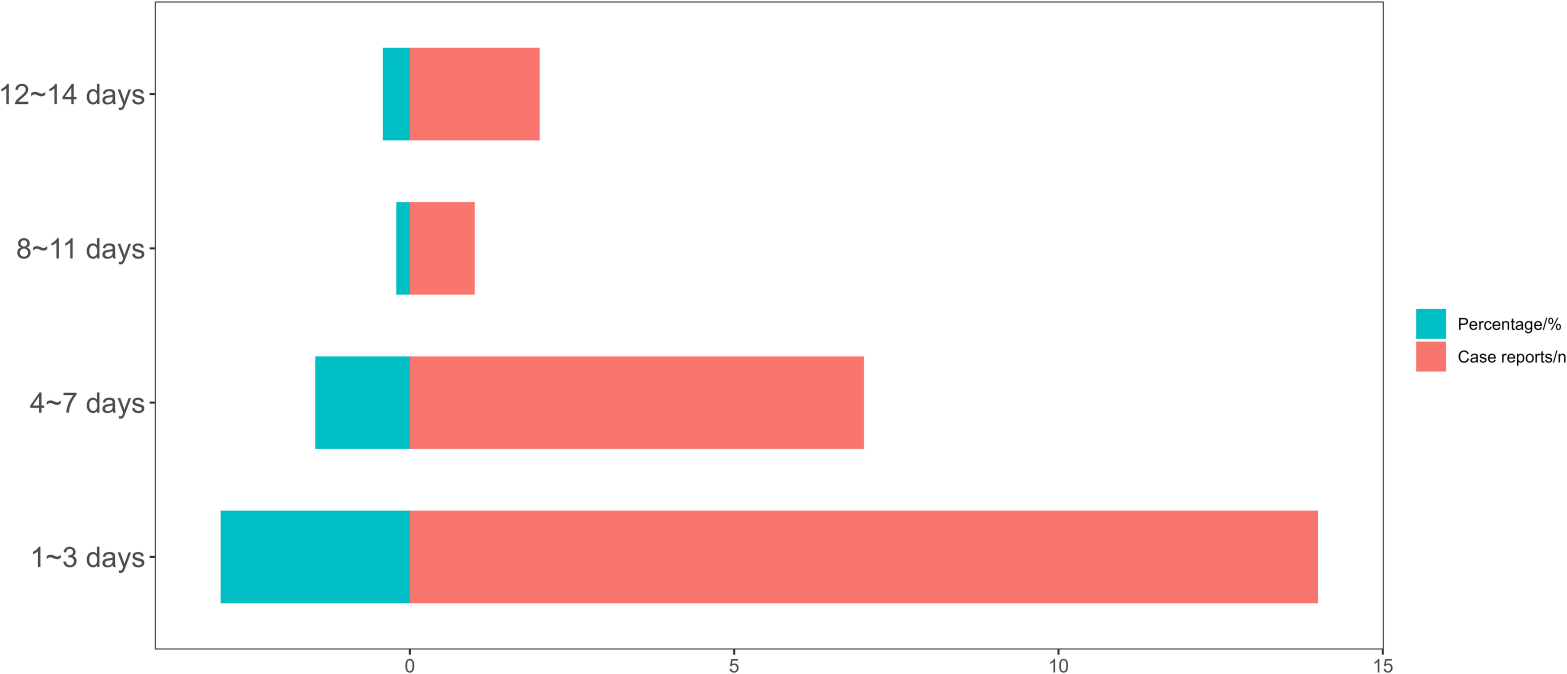
Time to onset of Zuranolone-related AEs.

The onset time of Zuranolone-related ADEs was collected from the database. After strict screening criteria, excluding cases with any incomplete data, a total of 24 reports (87 records at the PT level) were included in the analysis. The average onset time was 4 days, with a median onset time of 2 days (interquartile range [IQR] 1 to 6 days). As shown in **Figure 2**, the longest onset time was 14 days, which is consistent with the treatment course of Zuranolone. However, most cases occurred within the first 3 days of Zuranolone administration (n=14, 58.33%). The majority of cases occurred within the first week (n=21, 87.50%). However, there were a few cases with onset between 8 to 11 days (n=1, 4.17%) and 12 to 14 days (n=2, 8.33%), indicating that ADEs may occur at any time within two weeks. In addition, we listed the time series of adverse reactions by SOC classification and PT classification (**Table 6** and **Table 7**). In the PT classification table, we only selected recorded positive signals. Overall, General disorders and administration site conditions, Gastrointestinal disorders, Eye disorders, and other system organ diseases had faster onset times, while Nervous system disorders and Psychiatric disorders were slower. The remaining systems did not show a clear trend due to limited data.

**Table 6:** Time to onset of Zuranolone-related AEs at the SOC level.

System organ class	Case reports (n)	Mean onset time (d)	Maximum onset time (d)	Minimum onset time (d)
Nervous system disorders	26	3.3	1	13
Psychiatric disorders	26	5.0	1	14
General disorders and administration site conditions	13	2.5	1	6
Gastrointestinal disorders	5	1.2	1	2
Injury, poisoning and procedural complications	2	1.0	1	1
Musculoskeletal and connective tissue disorders	2	4.0	1	7
Eye disorders	2	1.0	1	1
Investigations	1	4.0	4	4
Respiratory, thoracic and mediastinal disorders	2	1.0	1	1
Reproductive system and breast disorders	3	2.0	2	2
Metabolism and nutrition disorders	2	6.0	1	11
Infections and infestations	1	1.0	1	1
Skin and subcutaneous tissue disorders	1	14.0	14	14
Cardiac disorders	1	3.0	3	3

**Table 7:** Time to onset of Zuranolone-related AEs at the PTs level.

Preferred terms	System organ class	Case reports (n)	Mean onset time (d)	Maximum onset time (d)	Minimum onset time (d)
Somnolence	Nervous system disorders	5	2.4	1	4
Dizziness	Nervous system disorders	7	2.7	1	7
Fatigue	General disorders and administration site conditions	2	1	1	1
Sedation	Nervous system disorders	2	9.5	6	13
Suicidal ideation	Psychiatric disorders	2	7.5	4	11
Tremor	Nervous system disorders	2	4	1	7
Feeling abnormal	General disorders and administration site conditions	3	3.7	2	6
Anxiety	Psychiatric disorders	3	5	2	11
Brain fog	Nervous system disorders	3	1	1	1
Insomnia	Psychiatric disorders	3	2.3	1	4
Feeling drunk	General disorders and administration site conditions	2	2	1	3
Depression	Psychiatric disorders	3	4.7	1	11
Vision blurred	Eye disorders	1	1	1	1
Migraine	Nervous system disorders	2	2.5	2	3
Memory impairment	Nervous system disorders	1	3	3	3
Muscle twitching	Musculoskeletal and connective tissue disorders	1	1	1	1
Nervousness	Psychiatric disorders	1	14	14	14
Cognitive disorder	Nervous system disorders	1	4	4	4
Initial insomnia	Psychiatric disorders	1	11	11	11
Bradyphrenia	Psychiatric disorders	1	7	7	7
Anger	Psychiatric disorders	1	3	3	3

### 3.4 Comparative reporting risk with other drugs against perinatal depression

**Figure 3:**
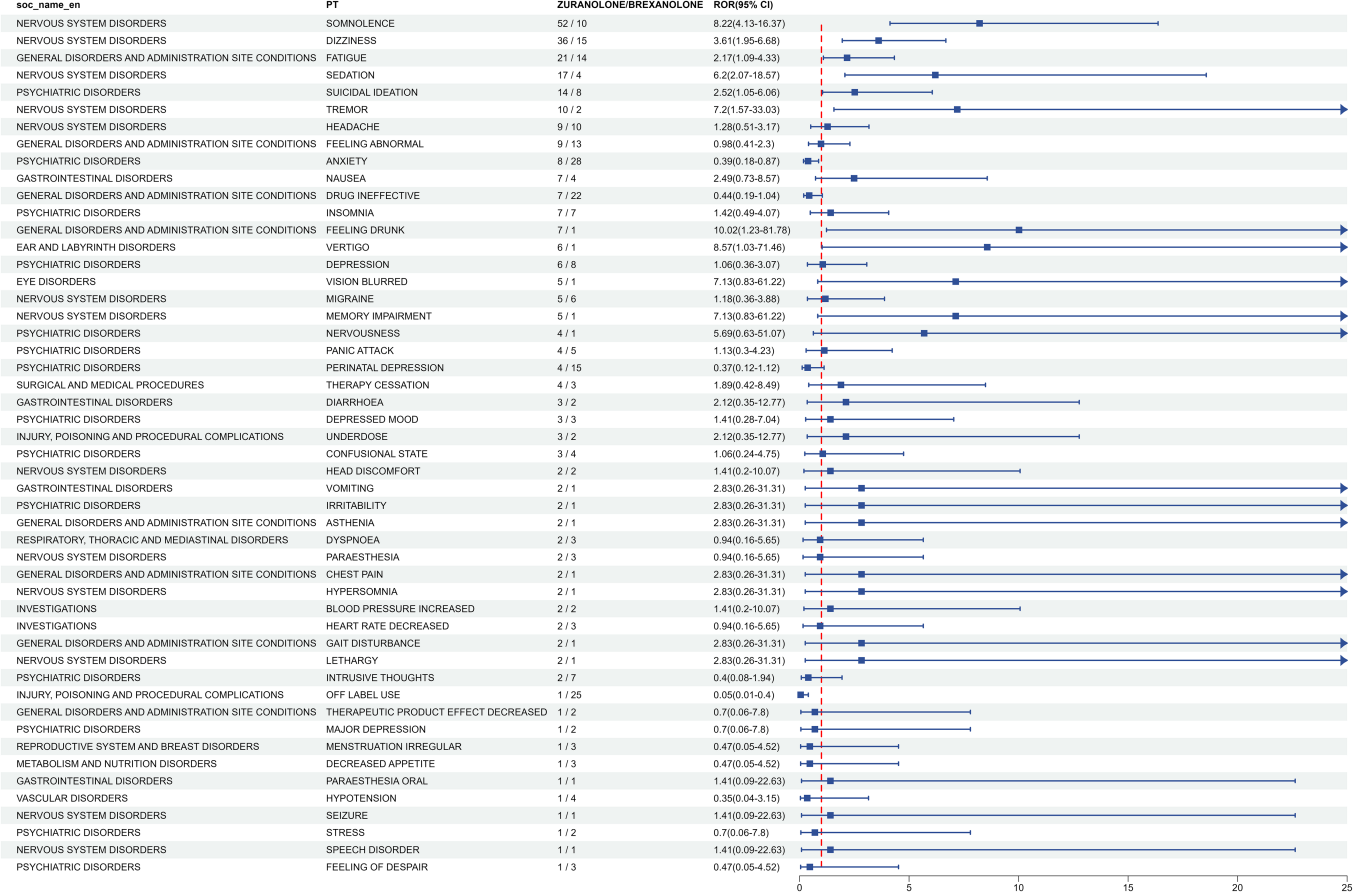
Analysis of Zuranolon-related ADRs compared with Brexanolone-related ADRs risk signals. Reporting odds ratios with 95% CI for top 50 ADEs.

Zuranolone, as an enhanced version of Brexanolone, which was marketed in 2019 for intravenous administration in adult patients with PPD and has the same mechanism of action, was compared to Brexanolone to analyze the association of adverse reactions between Zuranolone and Brexanolone (18). We used the ROR method to determine the top 50 incidence rates of common adverse reaction events between the two drugs that were disproportionately reported, and classified them by SOC. The results are shown in **Figure 3**. Some adverse reaction events, such as Somnolence, Dizziness, Fatigue, Sedation, Suicidal ideation, Tremor, feeling abnormal, Headache, and Nausea, were more common after Zuranolone administration. Brexanolone’s ADEs included product administration error, anxiety, off-label use, drug ineffective, drug monitoring procedure incorrectly performed, dizziness, perinatal depression, fatigue, product administration interrupted, feeling abnormal, crying, therapeutic product effect incomplete, incorrect product administration duration. But overall, Brexanolone’s adverse reactions were more related to psychiatric diseases, general diseases, and various reactions at the administration site and various injuries, poisoning, and procedural complications, while Zuranolone needs to pay more attention to Nervous system disorders and Psychiatric disorders.

## 4 Discussion

In previous studies, research on Zuranolone has primarily focused on its mechanism of action, literature reviews, and clinical trials, with few articles concentrating on the latest real-world data analysis. This comprehensive real-world pharmacovigilance study based on FAERS data describes the risk profile of adverse drug reaction reports following the market launch of Zuranolone, supplementing the assessment of its safety. Considering the treatment strategies for PPD, which often involve the combined use of therapeutic drugs, this article includes only reports where Zuranolone is the primary suspect drug to minimize related interference factors. The aim is to analyze the latest, meaningful adverse reactions to provide a basis for the rational use of drugs in clinical practice as soon as possible, achieving safer medication use.

In terms of the demographic and clinical characteristics of adverse events, the average age of occurrence for Zuranolone AEs is 31.6 years, which corresponds to the age distribution of the primary target population for Zuranolone. The main indication for Zuranolone is postpartum depression, which typically occurs in women of childbearing age; therefore, clinical studies and adverse reaction data focus on this age group. We found that perinatal depression is the most common indication, consistent with the intended use of Zuranolone. However, we identified one case in this batch of data where zuranolone was used to treat MDD. Due to the lack of efficacy data and concerns about tolerability, the drug has not been approved by the FDA for this indication. Therefore, this is clearly an off-label use, which requires widespread attention and emphasis in clinical practice, and adverse events caused by off-label use should be treated with caution. As demonstrated in the double-blind, randomized, placebo-controlled study, patients taking Zuranolone showed a statistically significant improvement in depressive symptoms, with a very rapid onset of action, showing clear effects by the third day (19). Moreover, at day 15, 87.6% of the Zuranolone-treated population maintained a response at day 42, proving the sustainability of its therapeutic effect. However, since Zuranolone is primarily used for the treatment of postpartum depression, patient gender was not specifically considered in this analysis. At the same time, we found that the serious outcomes reported for adverse reaction events associated with Zuranolone use include a very small number of life-threatening events, and some hospitalizations as well as other unspecified adverse events. Through retrieval of the Primary ID for these six patients, we identified adverse events associated with life-threatening outcomes and hospitalization. One life-threatening case report outcome was significantly linked to the occurrence of suicidal ideation, akathisia, and sedation. Additionally, adverse events including dizziness, major depression, panic attack, hypotension, decreased heart rate, neurological symptoms, blood pressure fluctuations, and syncope were associated with hospitalization. No fatal outcomes were observed in the available data, which may indicate a safer signal. As the clinical application of Zuranolone continues to increase, it is important for clinicians to be vigilant about AEs associated with Zuranolone, especially in younger patients. Early identification of AEs is necessary because these defects can be life-threatening or lead to the occurrence and progression of other diseases.

In our study, disproportionate signal mining results revealed Nervous system disorders and Psychiatric disorders as the most common system organ class adverse reactions, consistent with the mechanism of action of Zuranolone and the expected side effect profile. Zurzuvae is a neuroactive steroid allosteric modulator of the gamma-aminobutyric acid type A (GABA_A) receptor. The GABA system is the main inhibitory signaling pathway in the brain and central nervous system and plays an important role in regulating CNS function (20). Zurzuvae targets brain networks responsible for mood, arousal, behavior, and cognition functions. The GABA_A receptor is a pentameric structure composed of various combinations of subunits. Among these subunits, the α subunit, in particular, plays a crucial role in determining the functional properties of the GABA_A receptor (21). Different α subunits are known to mediate the sedative, hypnotic, and motor coordination effects of drugs. Cognitive impairment may stem from GABA_A receptors containing the α5 subunit. Preclinical characterization of zuranolone indicates that due to its selectivity for different GABA_A receptor subtypes, it may activate other receptor subtypes unrelated to therapeutic effects, thereby causing adverse reactions such as sedation and impaired motor function (22). Accumulated clinical data also indicate that Zuranolone has an acceptable safety profile and good tolerability compared to placebo (23). The central inhibitory adverse reactions indicated in the prescribing information for Zuranolone, such as somnolence, dizziness, and headache, are common in clinical practice. These adverse reaction events are usually mild and do not require discontinuation of treatment. However, moderate and severe adverse reaction events, such as suicidal ideation and behavior, cognitive and consciousness disorders, also account for a certain proportion in this study. Although less frequent in recorded serious adverse outcomes, clinicians should consider the exacerbation of PPD and be prepared to manage patients with emergent suicidal ideation and behavior after administering Zuranolone, including discontinuation of the drug. As its clinical application expands, continued pharmacovigilance is crucial for clarifying the comprehensive safety profile of Zuranolone.

However, the identification of Ear and labyrinth disorders as an unexpected positive signal, not mentioned in the prescribing information, may indicate a new safety issue that requires further research for validation. The mechanism underlying the otic and labyrinthine adverse reactions of Zuranolone is not yet clear, and these adverse reactions may stem from the drug’s effects on the central nervous system and vestibular function. Vertigo occurs due to vestibular compensation disorders, inducing static vestibular symptoms (such as spontaneous nystagmus and postural deviation) and dynamic vestibular symptoms (such as oscillopsia and an ataxic gait) (24). Neurosteroids increase in concentration under stress conditions and produce behavioral effects. Electrophysiological studies have determined that the neurosteroid dehydroepiandrosterone sulfate inhibits GABAergic inhibition of medial vestibular nucleus neurons through GABA_A receptors, which may lead to a disruption in the control of vestibular nucleus neuron activity and thus contribute to the development of vertigo (25). GABA_A receptors are widely distributed in the central nervous system and vestibular neurons, playing an important role in regulating the excitability of vestibular nuclear neurons (26). In the mammalian labyrinth, the GABA_A receptor subtype is involved in the excitatory neurotransmission between vestibular type II hair cells and afferent neurons (27). When Zuranolone promotes the activity of these receptors, it can lead to significant changes in neuronal excitability, thereby affecting vestibular input and integration, and subsequently interfering with the processing of vestibular information. However, this is merely speculation, and the specific mechanism still requires further basic experimental research for confirmation. Unfortunately, there is currently no relevant research in this area. In addition, the impact of Zuranolone on systemic hemodynamics may also be a potential mechanism for inducing ear and labyrinth-related adverse reactions. Further clinical and basic research is necessary to delve into the effects of Zuranolone on vestibular function and its potential biomarkers to optimize its clinical application and reduce the incidence of adverse reactions.

Among the AEs mentioned in the prescribing information, Fatigue, Feeling abnormal, Underdose, and Therapy cessation all reached significant statistical signal strength. Among the adverse reactions not mentioned in the instructions, Vision blurred is particularly noteworthy. The ocular adverse reactions of Zuranolone may be related to its impact on retinal neural transmission. The retina is the first station for visual information processing, and factors affecting its function may lead to blurred vision (28). Moreover, the inhibitory effect of Zuranolone on neuroinflammation may indirectly affect retinal blood flow and metabolism, which requires attention in clinical application (29). GABA receptors are associated with the regulation of ocular growth and refraction, and GABA_A is an ionotropic receptor, which is the main GABA receptor subtype located in retinal cells (30). As expected, agonists and antagonists of the GABA_A receptor subtype have been found to have the most potent effects in the context of form deprivation myopia (FDM), including both axial elongation and inhibition. GABA_A agonists reduce the release of dopamine (DA) in the retina, and have an additive effect on the protective role of inhibiting normal vision in the occurrence of FDM. The protective effect of normal vision against form deprivation can be altered simultaneously through the GABA and DA pathways (31). Therefore, Zuranolone, as a neuroactive steroid GABA_A receptor positive allosteric modulator, may cause adverse effects related to the eyes and vision through this pathway. Future research should further explore its mechanism of action and assess its safety and efficacy in different patient populations.

AEs involving the respiratory system, digestive system, and Metabolism and nutrition disorders are still worth noting, even though their signal strength did not reach significant statistical significance. We should pay attention to whether they have clinical significance in guiding clinical drug therapy. The respiratory and digestive system adverse reactions mentioned in Zuranolone’s summary of product characteristics (SmPC) include dyspnea, yawning, discomfort in the mouth and throat, nausea and vomiting, and diarrhea, etc. Adverse reactions of the reproductive system and breast diseases such as Heavy menstrual bleeding, Polymenorrhoea, Dysmenorrhoea, Galactostasis, and Menstruation irregular, along with the fetal toxicity emphasized in Zuranolone’s prescribing information, highlight the significant impact of Zuranolone on pregnant women and fetuses (32). Clinicians should inform pregnant women and women of childbearing age about the potential damage to their reproductive system and the potential risks to the fetus in the womb after taking the medication. In the analysis of the onset time of adverse reactions, we found that General disorders and administration site conditions, Gastrointestinal disorders, Eye disorders, and other system organ diseases have a faster onset of adverse reactions, while Nervous system disorders and Psychiatric disorders are slower. Overall, the distribution of the onset time of Zuranolone-related AEs has a certain regularity. Most AEs appear early in the medication, which may be related to the pharmacological mechanism of the drug, but the accuracy of this data may be affected by factors such as a small sample size, but it is also worthy of attention from clinical medical staff.

Our study has some limitations to consider. First, our source of information is a widely used spontaneous reporting system database, which has its inherent limitations, such as population bias, underreporting, missing information, duplicate reports, and inconsistent formats. Second, disproportionate analysis cannot completely eliminate the methodological confounding factors of concomitant medications, and further clinical case-control studies are needed (33). Moreover, the involvement of non-physician reporters may introduce detection bias, particularly for complex drug-event associations that require professional expertise. Therefore, we also hope that more clinicians will report relevant adverse events to the FDA in the future to enrich real-world evidence. However, despite the limitations of the FAERS database in pharmacovigilance research, the comprehensive characterization of Zuranolone’s AE signals and the discovery of some unexpected AE signals may provide a basis for further clinical research on Zuranolone, as well as more comprehensive and updated evidence for the clinical safety of Zuranolone. In addition, it also provides some preventive medication warnings for more countries where Zuranolone has not yet been marketed. The safety profile of Zuranolone still requires continuous monitoring.

The comprehensive characterization of these AE signals provides some evidence-based basis for optimizing the clinical practice of zuranolone:(1) We recommend that clinical practice needs to establish a pre-treatment risk assessment template, incorporating a pre-treatment baseline assessment of the risk of some high-risk or fatal adverse events, such as suicide risk screening and vestibular function assessment; (2) Formulate a phased monitoring plan, emphasizing key monitoring nodes. Not only should we focus on the risk of adverse events occurring during the early stage of treatment or the peak period of blood drug concentration, but also the maintenance treatment period and the period with a high incidence of withdrawal reactions should be treated with caution. Meanwhile, we can refer to the time intervals for different adverse events; (3) Clinical attention during medication use should not only focus on the monitoring of physiological indicators, but also continuously assess the patient’s psychological state and mental symptoms through psychological scales and other methods; (4) Future research needs to improve guidelines for the use of special populations, especially for patients with liver and kidney dysfunction and the elderly group. The implementation of these specific measures will help clinical doctors use the drug more safely, and it is also recommended that drug regulatory agencies establish a comprehensive and extensive adverse reaction registration system to continuously collect long-term safety data.

## 5 Conclusion

In the post-marketing study, our pharmacovigilance study reported providing adverse events after Zuranolone medication. In conclusion, our findings may promote clinical awareness of Zuranolone-related toxicity and help health care professionals to mitigate the risk of adverse events.

## Data Availability

The original contributions presented in the study are included in the article/**Supplementary Material**. Further inquiries can be directed to the corresponding author.
